# Surface modifications to promote the osteoconductivity of ultra‐high‐molecular‐weight‐polyethylene fabrics for a novel biomimetic artificial disc prosthesis: An in vitro study

**DOI:** 10.1002/jbm.b.35163

**Published:** 2022-09-16

**Authors:** Celien A. M. Jacobs, Esther E. A. Cramer, Aylvin A. Dias, Harold Smelt, Sandra Hofmann, Keita Ito

**Affiliations:** ^1^ Orthopaedic Biomechanics, Department of Biomedical Engineering Eindhoven University of Technology Eindhoven The Netherlands; ^2^ Institute for Complex Molecular Systems, Eindhoven University of Technology Eindhoven The Netherlands; ^3^ DSM Biomedical Sittard‐Geleen The Netherlands

**Keywords:** bone ingrowth, in vitro, polyethylene (UHMWPE), spinal implant, surface modification

## Abstract

A novel biomimetic artificial intervertebral disc (bioAID) for the cervical spine was developed, containing a hydrogel core representing the nucleus pulposus, an UHMWPE fiber jacket as annulus fibrosis, and titanium endplates with pins for mechanical fixation. Osseointegration of the UHMWPE fibers to adjacent bone structures is required to achieve proper biomimetic behavior and to provide long‐term stability. Therefore, the aim of this study was to assess the osteoconductivity of several surface modifications of UHMWPE fabrics, 2D weft‐knitted, using non‐treated UHMWPE fibers (N), plasma treated UHMWPE fibers (PT), 10% hydroxy apatite (HA) loaded UHMWPE fibers (10%HA), plasma treated 10%HA UHMWPE fibers (PT‐10%HA), 15%HA loaded UHMWPE fibers (15%HA) and plasma treated 15%HA UHMWPE fibers (PT‐15%HA). Scanning electron microscopy (SEM) was used for surface characterization. Biological effects were assessed by evaluating initial cell attachment (SEM, DNA content), metabolic activity (PrestoBlue assay), proliferation, differentiation (alkaline phosphatase activity) and mineralization (energy dispersive x‐ray, EDX analysis) using human bone marrow stromal cells. Plasma treated samples showed increased initial cell attachment, indicating the importance of hydrophilicity for cell attachment. However, incorporation only of HA or plasma treatment alone was not sufficient to result in upregulated alkaline phosphatase activity (ALP) activity. Combining HA loaded fibers with plasma treatment showed a combined effect, leading to increased cell attachment and upregulated ALP activity. Based on these results, combination of HA loaded UHMWPE fibers and plasma treatment provided the most promising fabric surface for facilitating bone ingrowth.

## 1 INTRODUCTION

**FIGURE 1:**
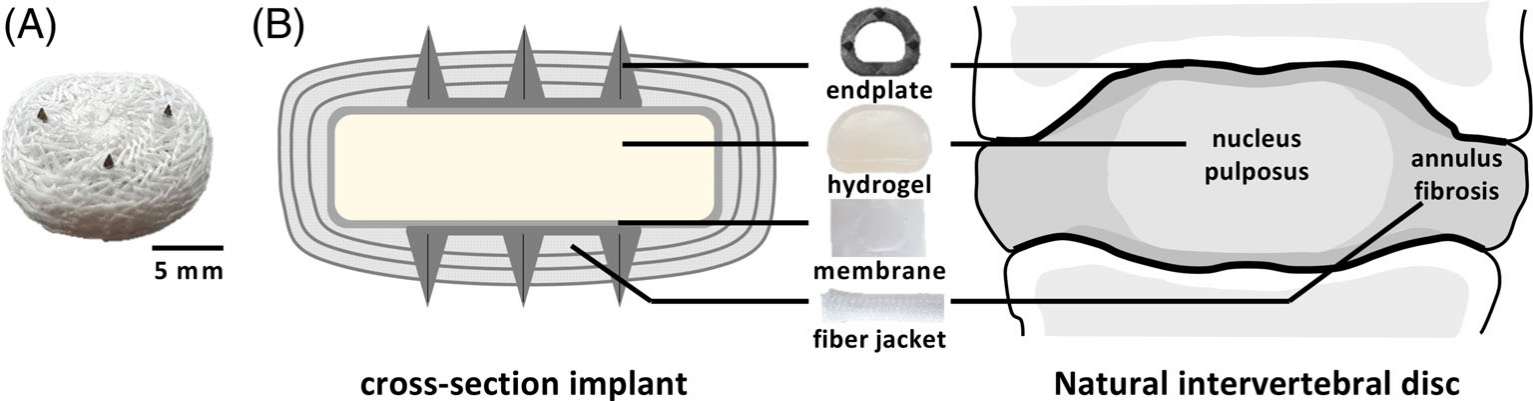
(A) Biomimetic artificial intervertebral disc (bioAID). (B) Schematic representation of the design of the bioAID and its biomimicry compared to a natural disc

Cervical artificial intervertebral discs (AIDs) have been developed as a mobility preserving alternative treatment for severely degenerated discs. First generation prostheses were based on traditional synovial joint articulating arthroplasty designs, leading to a mismatch in the motion and kinematics of a natural cervical disc.^1^, ^2^ This mismatch could potentially lead to a hypermobile environment where other anatomical structures need to compensate for this altered loading regime in the spine. It is therefore hypothesized that mimicking the native structure of the cervical intervertebral disc (IVD) would also lead to natural biomechanical properties. As a result, second generation prosthesis have been developed in recent years, that aim to better replicate the anatomy of a natural disc.^3^, ^4^, ^5^, ^6^, ^7^ One of those is the novel biomimetic cervical AID developed by Peter van den Broek (Figure 1).^7^ The design contains a hydrogel core, representing the swelling nucleus pulposus enclosed in a ultra‐high‐molecular‐weight‐polyethylene (UHMWPE) fiber jacket mimicking the annulus fibrosus.^8^ Although titanium endplates with pins are used to achieve initial stabilization to the vertebrae, direct anchorage or osseointegration of the UHMWPE fibers to the adjacent bone structures is required to achieve proper biomimetic behavior.^9^ Moreover, osseointegration is crucial to provide long‐term stability, being one of the most important factors influencing clinical success of load bearing prostheses.^10^ Although it has good mechanical properties, the disadvantage of pure UHMWPE is that it is inert and hydrophobic, making it less attractive for cells and proteins to attach and facilitate osseointegration.^11^

To increase the osteoconductivity, defined as a material surface that facilitates bone ingrowth, implant surfaces are often chemically or physically altered.^12^ One common approach to increase osteoconductivity is to increase surface roughness. High surface roughness is known to stimulate cell differentiation towards osteoblasts, allow for better biomechanical connection and lead to more bonding spots for host proteins to interconnect with the implant surface.^13^, ^14^ Several methods to increase surface roughness are sand blasting, etching or oxidation.^14^, ^15^, ^16^ Besides surface roughness, an increased hydrophilicity has also shown to promote cell attachment in vivo and in vitro and thereby promote osseointegration.^17^, ^18^, ^19^, ^20^, ^21^ Increasing hydrophilicity of the surface can be achieved by methods such as plasma treatment and UV irradiation. The implant surface can also be chemically altered by applying calcium phosphate (CaP) and hydroxyapatite (HA) coatings, which allow for a chemical bonding between the implant and bone surfaces due to the chemical similarities of bone and CaP and HA.^22^, ^23^ Several in vivo studies have shown an increase in bone ingrowth for implants coated with HA or CaP.^24^, ^25^ However, there are also concerns with plasma‐sprayed coatings. Some studies have shown there is a risk of delamination of the coating from the surface of the implant, resulting in clinical implant failure due to the micromotion caused by debris and loose particles.^26^, ^27^ As a solution, incorporation of HA into the material has shown to be beneficial in providing a mechanically stable surface for facilitating bone ingrowth.^28^

Incorporation of ceramics, such as HA, into polymeric materials to increase osteoconductivity is mainly reported for solid surfaces, since spinning of composite materials into fibers is challenging.^29^ Fiber production from composite materials can lead to instabilities and frequent breakage during the gel spinning process and unwanted alterations in the bulk mechanical properties. Another potential problem is that the added ceramic particles are often unavailable for biological interaction since most of the particles are covered by the polymeric material due to the production process. In the current article, a novel fiber is introduced that is gel spun out of a composite solution containing UHMWPE and HA. These novel fibers have bioactive surfaces while preserving the desired fiber mechanical properties for orthopedic applications. To increase the exposure of the HA particles at the fiber surface, additional surface treatments can be performed, such as an etching step with plasma.

To date, few studies can be found on physically or chemically altered UHMWPE fabrics used for orthopedic/spinal applications. Most studies have investigated physically or chemically altered metal, since polymers are often avoided at the bone implant interface due to the lower affinity for bone ingrowth.^30^ To our knowledge, only one other spinal implant (3D‐F) used UHMWPE fibers at the bone‐implant surface spray coated with sintered HA or apatite wollastonite glass ceramics granules to increase osteoconductivity.^31^, ^32^, ^33^, ^34^, ^35^ Initial in vivo data showed penetration of scar tissue into the fabric and loss of bioceramic micropowders after implantation.^33^ In the following study, in vivo results showed that the fibers were directly surrounded by osseous trabeculae^31^ and, that the implant was firmly fixed to the vertebral body only when implanted in a stable environment.^32^

**FIGURE 2:**
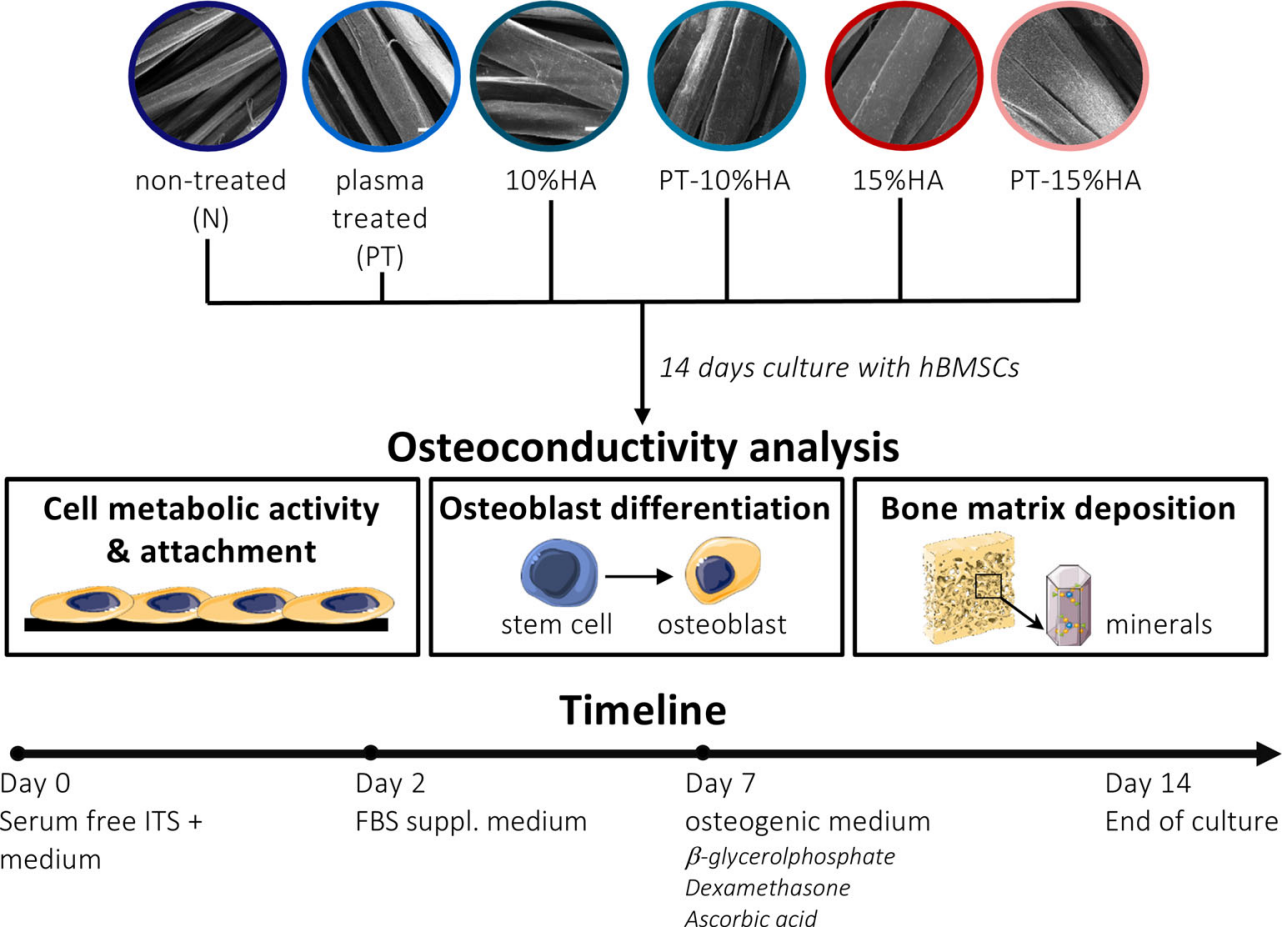
Schematic representation of experimental set‐up. Weft‐knitted fabrics made of untreated (N), plasma treated (PT), 10%HA loaded (10%HA), plasma treated 10%HA loaded (PT‐10%HA), 15%HA loaded and, plasma treated 15%HA loaded UHMWPE fibers (PT‐15%HA) were cultured with human bone marrow‐derived stromal cells (hBMSCs) for 14 days. Osteoconductivity was assessed based on cell viability, attachment, osteogenic differentiation, and mineral deposition. Image created with Servier Medical Art in compliance with the terms of the Creative Commons Attribution 3.0 Unported License

Due to the limited data available, it is important to get an indication which surface modifications are most suitable in facilitating osseointegration of the UHMWPE fabric surface of the bioAID. Therefore, this study aimed to assess the initial in vitro osteoconductive response of UHMWPE fabrics by modifying the surface roughness, hydrophilicity and/or incorporation of HA particles into the fiber. In the present study, an osteoconductive material is defined as a material that facilitates bone growth on its surface. New tissue formation on a material is mainly promoted by a surface structure that facilitates cell adherence, cell proliferation and production of extracellular matrix. As a result, osteoconductivity was graded based on three characteristics: cell metabolic activity and attachment, osteoblast differentiation and bone matrix production. Human Bone Marrow Stromal cells (hBMSC) were seeded on weft‐knitted UHMWPE fabrics in vitro to assess the osteoconductive potential of these different surfaces (Figure 2).

## 2 MATERIALS AND METHODS

### 2.1 Fabric sample preparation

Six differently modified 2‐dimensional (2D) weft‐knitted fabrics were prepared: non‐treated UHMWPE fibers (N), plasma treated UHMWPE fibers (PT), 10%HA loaded UHMWPE fibers (10%HA), Plasma treated 10%HA UHMWPE fibers (PT‐10%HA), 15%HA loaded UHMWPE fibers (15%HA) and plasma treated 15%HA UHMWPE fibers (PT‐15%HA). Preparation of the 10% and 15%HA loaded biocomposite UHMWPE fibers was performed using a gel spinning process as described in Dias et al. (2021) and provided by DSM Biomedical, Geleen.^36^ Next, untreated UHMWPE fibers (Dyneema Purity® SGX dtex100 TS 100, DSM, Geleen, Netherlands), 10%HA UHMWPE and 15%HA UHMWPE fibers g/m were weft‐knitted (Shima Seiki 13 gauge knitting machine) into 2D fabrics using standard large hook needles (GCN 1053A, Groz‐Beckert) with areal densities of 75–80 g/m^2^. Depending on the experimental group, select samples were prepared for plasma treatment by winding them around the drum electrode (along the cylinder circumference of 1.8 m) of a pilot‐scale web coater. Plasma treatment was performed with argon and oxygen for 40 min (40 min, Ar/0, plasma. 160/40 sccm. 1000 W, I mbar), resulting in an etching rate of about 25 nm/min. The selected pressure of 1 mbar supports plasma‐chemical etching involving rather long‐living oxygen species that diffuse along the fibrous surfaces. After surface treatments, all fabrics were cut into circular pieces with a diameter of 9.5 mm. All fabric discs were sterilized by incubation in 0.5 ml isopropanol for 1 hour, followed by evaporation and washing twice for 5 min with 0.5 ml PBS (Dulbecco's Phosphate Buffered Saline, Sigma Aldrich).

### 2.2 Materials

Dulbecco's modified Eagle medium (DMEM), non‐essential amino acids (NEAA) and antibiotic/antimycotic (Anti‐Anti) were from Life Technologies. Trypsin was from Lonza. Bovogen fetal bovine serum (FBS) was from PAA laboratories (FBS Gold). Dexamethasone, ascorbic acid‐2‐phosphate and β‐glycerolphosphate were obtained from Sigma Aldrich, ITS+ from Corning (Fisher Scientific).

### 2.3 Cell culture

Human Bone Marrow‐derived Stromal Cells (hBMSCs) isolation and characterization from human bone marrow (Lonza) was performed as previously described.^37^ Passage five hBMSCs were expanded in expansion medium (DMEM; Cat. No. 41966, 10% FBS, 1% anti‐anti, 1% NEAA). After the cells reached confluency (day 7), the cells were trypsinized, counted and centrifuged. To prepare cell‐laden constructs, hBMSCs were suspended in seeding medium (DMEM, 1%ITS+, 1% anti‐anti) at a density of 400,000 cells/ml. To ensure that cells only attached to fabric constructs and not to the well, cell repellent 48 wells plates were used (Cellstar®, Greiner Bio One). To prevent the fabric constructs from floating, silicon O‐rings (Technirub, O‐ring, 9 × 2 silicone 70 shore rood) were put on top of each fabric construct. Next, the cells were drop seeded on the fabric constructs (20,000 cells/50 μl) and incubated for 4.5 h at 37°C, 5%CO_2_ to allow for cell attachment before cells were completely submerged with seeding medium with a total volume of 0.5 ml per well. At day 2, 1%ITS+ was replaced with 10% Bovogen FBS in the medium. On day 7, medium was supplemented with osteogenic supplements (50 μg/ml L‐ascorbic‐acid‐2‐phosphate, 100 nM dexamethasone and 10 mM β‐glycerophosphate). Samples were cultured for 14 days at 37°C, 5% CO_2_, medium was changed every 3 days.

### 2.4 Cell metabolic activity, DNA content and alkaline phosphatase activity

To determine metabolic activity of all viable cells, a non‐destructive PrestoBlue assay (*n* = 6 per group) was performed at day 2, 7, and 14. PrestoBlue (Thermo Fisher Inc.) was added to each well (10% v/v), including a blank with only medium, and incubated at 37°C, 5% CO_2_ for 30 min. After incubation, the fluorescence was measured with excitation wavelength at 530–560 nm and emission wavelength at 590 nm with a plate reader (Synergy™ HT, BioTek® Instruments Inc.). The measured fluorescence was corrected for the blank, and results are presented as fluorescence intensity. Next, samples were washed in PBS (Dulbecco's Phosphate Buffered Saline, Sigma Aldrich) before performing alkaline phosphatase activity (ALP) assay (day 14) and/or DNA assay (day 2, 7, and 14). For the ALP assay, samples were disintegrated in 0.5 ml of 0.2% (v/v) Triton X‐100 and 5 mM MgCl_2_ solution using steel beads and a MiniBeadbeater™ (Biospec). After centrifugation at 3000 g for 10 min, the supernatant (80 μl) was combined with 0.75 M 2‐amino‐2methyl‐1‐propanol (AMP) buffer solution (20 μl) and 10 mM p‐nitrophenylphosphate substrate solution (100 μl). This was incubated until a color change was observed (±10 min), then 100 μl of 0.2 M NaOH was added to stop the conversion of p‐nitrophenyl phosphate into p‐nitrophenol. Absorbance values were determined at 405 nm with a microplate reader (Synergy™ HT, BioTek® Instruments Inc.). Absorption values were subtracted from the measured blank. ALP activity was calculated using a standard curve obtained from samples with known p‐nitrophenol concentrations, ranging between 0 and 0.9 mmol/ml. The calculated ALP activity was then normalized by the DNA content measured for each construct. Samples for DNA assessment were digested using papain (125 μg/ml) and DNA content was determined by using Qubit™ dsDNA HS Assay Kit (Invitrogen), according to the manufacturer's instructions.

### 2.5 Scanning electron microscopy/energy dispersive x‐ray

After 14 days of culture, the cell loaded samples were fixed with 2.5% glutaraldehyde (Sigma Aldrich) in 0.1 M Sodium Cacodylate (Sigma Aldrich) buffer for 15 min and washed with 0.1 M Sodium Cacodylate buffer. Control samples without cells and cell loaded samples were dehydrated using 0.5 ml of multiple ethanol series (50% twice, 70% twice, 95% twice, 100% three times for 10 min) and were dried chemically with hexamethyldisilazane (Sigma Aldrich). Then the samples were washed three times with 0.5 ml ultrapure water for 5 min, dried by air and mounted on specimen stubs. To provide better contrast, only the samples for scanning electron microscopy (SEM) imaging were sputter coated with 8 nm gold (Q3150T, Quorum Technologies). SEM images were obtained using a Quanta 600 SEM (Thermo Scientific Breda, The Netherlands), in a high vacuum (<1.3 × 10^−4^) at 10 kV with a spot size of 3 using the Everhart‐Thornley secondary electron detector (ETD‐SE). Similar sample preparation was utilized to perform energy dispersive x‐ray (EDX) (Phenom ProX Desktop, ThermoFisher) analysis to evaluate regions in which extracellular matrix depositions were identified (10 kV, backscattered electron detector).

### 2.6 Statistical analysis

Mean and standard deviation were calculated using Microsoft Excel. Comparisons between experimental groups were determined by one‐way ANOVA, followed by Tukey's honest post‐hoc analysis to determine significant differences. Normal distribution was evaluated using Shapiro–Wilk test and homogeneity of variances was assessed using Levene's test. If the experimental groups did not show homogeneity of variances but had a normal distribution, Welch and Brown‐Forsythe ANOVA was used. In all cases *p* < .05 was considered as statistically significant. Statistical comparisons between the experiment groups were performed with GraphPad Prism version 8.0.2 for Windows (GraphPad Software).

## 3 RESULTS

### 3.1 Surface characterization

#### 3.1.1 Surface topography by SEM

**FIGURE 3:**
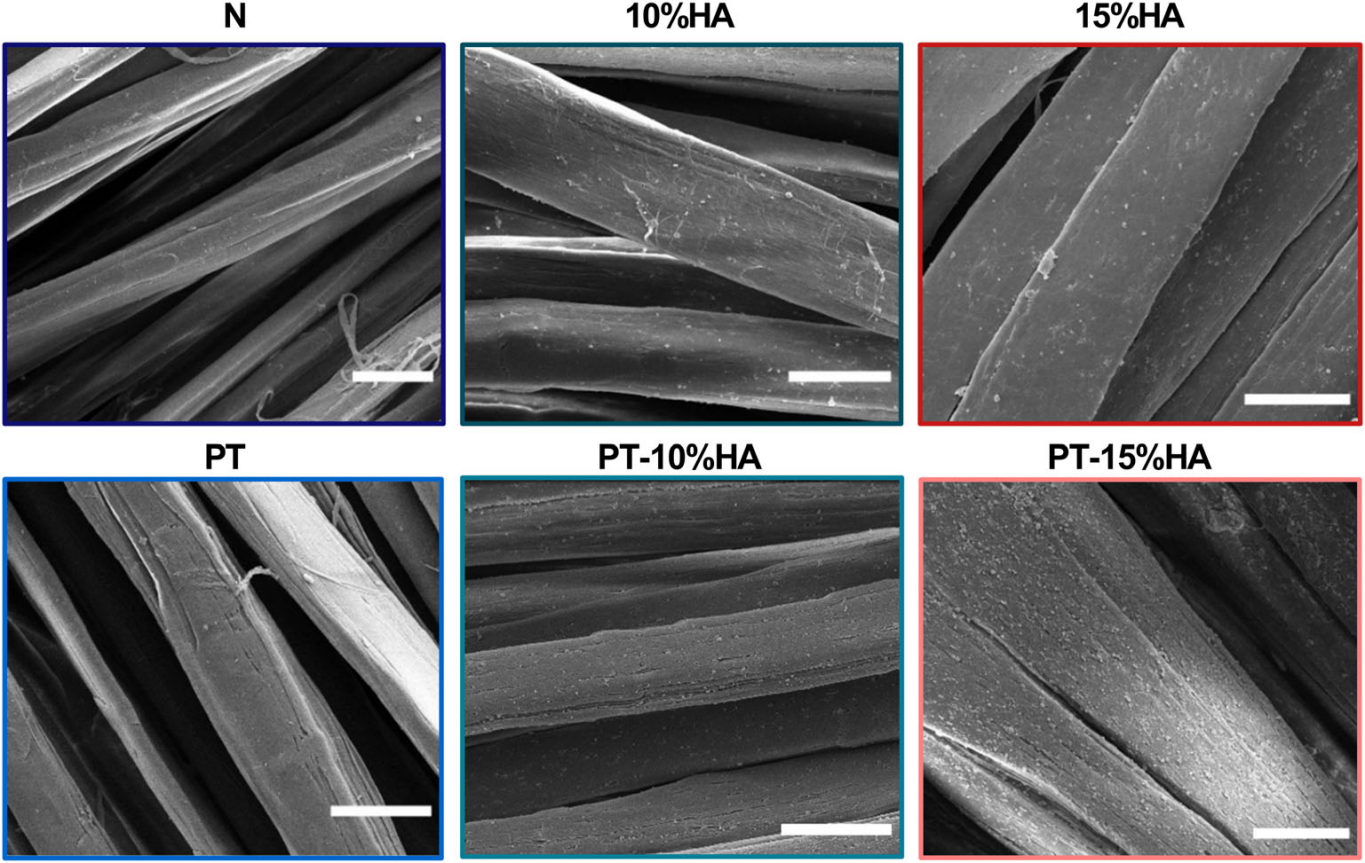
Surface characterization by scanning electron microscopy of the untreated (N), plasma treated (PT), 10%HA loaded (10%HA), plasma treated 10%HA loaded (PT‐10%HA), 15%loaded and, plasma treated 15%HA loaded fabrics (PT‐15%HA). Scale bar: 25 μm

SEM micrographs showed the presence of HA particles in the 10%HA, 15%HA, PT‐10%HA and PT‐15%HA groups, while smooth plain fibers were observed for the N and PT group (Figure 3). Application of plasma treatment to the 10%HA and 15%HA fabrics resulted in an increased amount of HA particles being exposed at the surface compared with non‐plasma treated fibers of same composition. As a result, the surface roughness also increased due to the increased particles exposed at the surface (only microscopically observed and not quantified). No visible difference in amount of HA particles was microscopically observed between 10%HA and 15%HA. However, more HA particles seem to be exposed on the surface for the PT‐15%HA compared with the PT‐10%HA surfaces.

### 3.2 In vitro biological response

#### 3.2.1 Cell attachment

**FIGURE 4:**
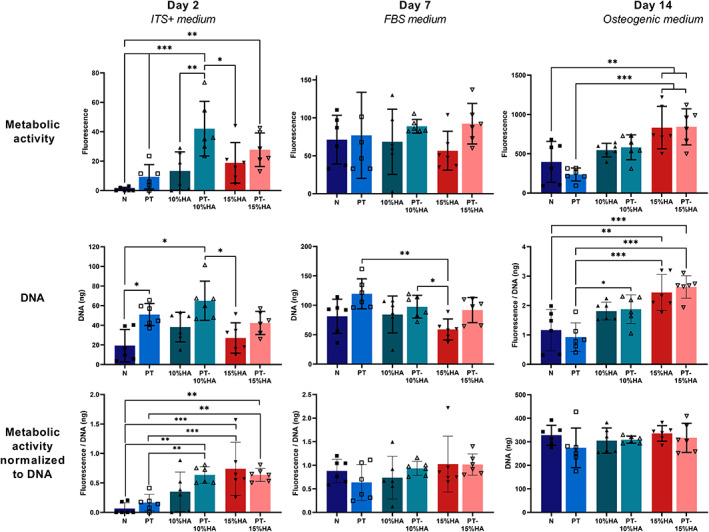
Metabolic activity (Fluorescence, mean ± SD) measured by PrestoBlue assay on day 2, 7, and 14. DNA content (ng, mean ± SD) on day 2 showing initial cell adherence and on day 7 and 14 to assess proliferation over time. Metabolic activity normalized for DNA content (fluorescence/ng, mean ± SD) on day 2,7 and 14. One way ANOVA (*p < .05; **p < .01; ***p < .001)

Cell attachment was assessed to determine the affinity of hBMSCs to adhere to the material surface. Applying plasma treatment increased the mean initial amount of DNA and overall metabolic activity compared with the same fiber composition without plasma treatment (Figure 4, Day 2). However, only PT and PT‐10%HA showed a significantly higher amount of DNA compared with the untreated group, while for the overall metabolic activity this held true for all plasma treated groups in comparison to the untreated fabrics.

**FIGURE 5:**
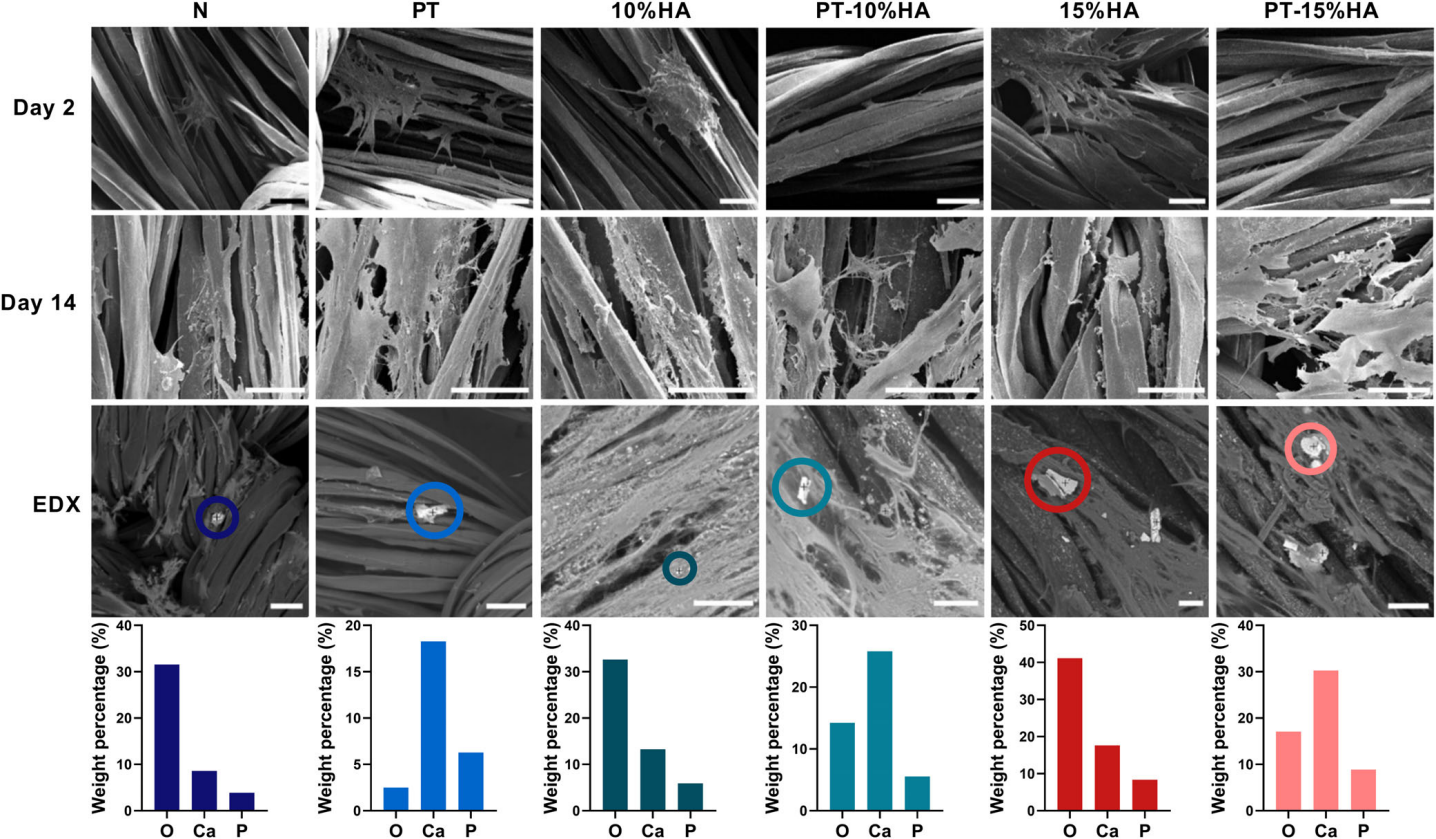
Scanning electron microscopy images of the differently modified UHMWPE surfaces (N: untreated, PT: plasma treated, 10%HA: 10% hydroxyapatite loaded, PT‐10%HA: plasma treated 10%HA loaded, 15%HA: 15% hydroxyapatite loaded, PT‐15%HA: plasma treated 15%HA loaded) on day 2 to visualize cell adherence and cell morphology and on day 14 to show cell morphology and matrix deposition. Representative energy dispersive x‐ray spot analysis of matrix minerals for all groups on day 14 showing the elements of which the spots are composed of. Scale: 50 μm

SEM images on day 2 (Figure 5) verified the attachment of cells on surfaces in all experimental groups. Adhered hBMSCs showed a similar flattened and spread morphology with numerous filopodia on all surfaces.

#### 3.2.2 Cell metabolic activity and proliferation

The normalized metabolic activity on day 2 shows increased activity with the presence of HA particles in the fiber. When looking at the proliferation, over time, a large increase in DNA content and metabolic activity was observed for all groups (Figure 4, day 7 and 14). This is also verified with the increased cell density visible on SEM images on day 14 for all samples (Figure 5). On day 7, the effect of plasma treatment on increased DNA content and metabolic activity seemed to disappear. On day 14, also no significant difference in DNA content was observed. However, differences in metabolic activity were still present, showing increased mean metabolic activity (overall and normalized to DNA) with increasing HA content.

#### 3.2.3 Osteogenic differentiation

**FIGURE 6:**
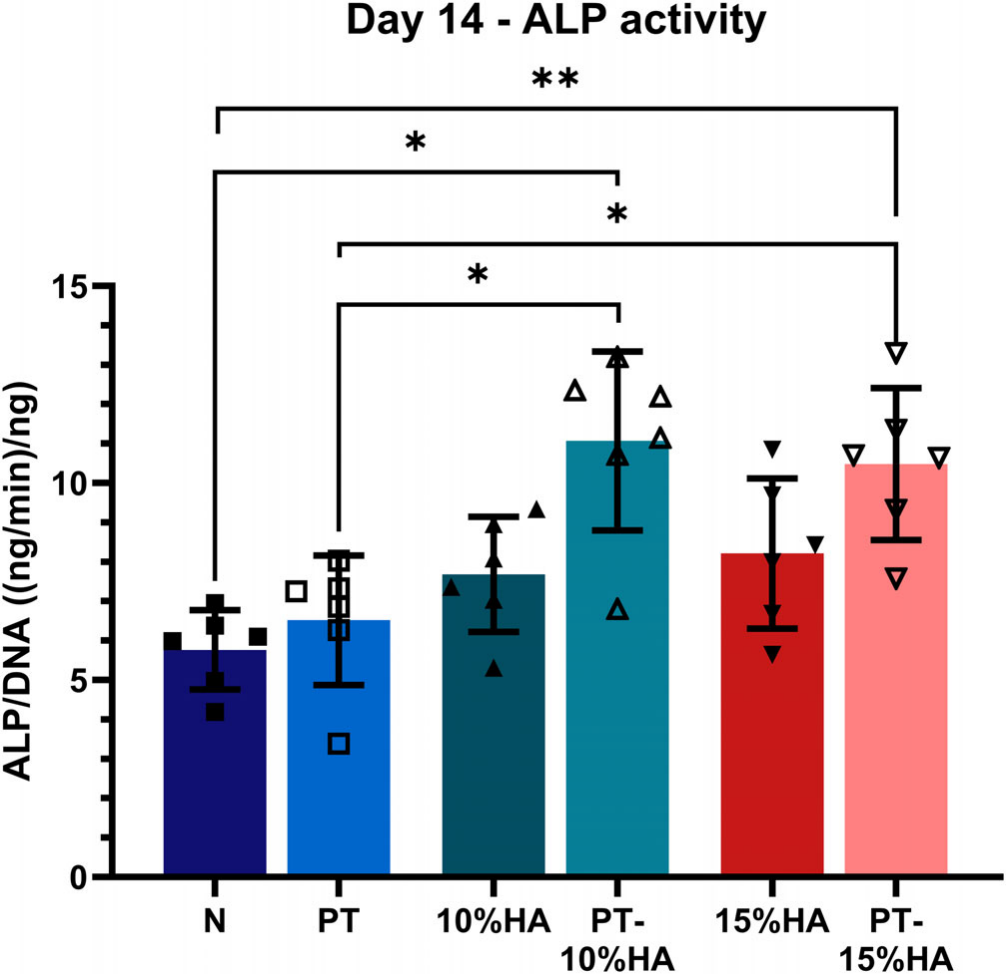
Alkaline phosphatase activity normalized for DNA content as a marker for osteogenic differentiation ([ng/min]/ng, mean ± SD) on day 14. One way ANOVA (*p < .05; **p < .01; ***p < .001)

ALP activity is often used as an osteoblastic differentiation marker in in vitro experiments.^38^ At day 14, the HA loaded and plasma treated fibers (PT‐10%HA, PT‐15%HA) showed significantly higher ALP activity per cell compared with both the untreated (N) and plasma treated (PT) group (Figure 6).

#### 3.2.4 Extracellular matrix deposition

SEM images showed matrix deposition in all groups (Figure 5). EDX analysis also confirmed the presence of calcium and phosphorus elements, being the major components of calcium phosphate apatite, forming the mineral phase of bone (Figure 5). EDX was only used as a method to identify the elements of the nodules, not to evaluate the differences in weight percentage between the groups. However, more nodules containing calcium and phosphate were detected for the HA loaded fibers (10%HA, PT‐10%HA, 15%HA and PT‐15%HA) and PT group compared with the untreated group (N).

## 4 DISCUSSION

Osseointegration between an implant and the bone surface is of great importance to provide stability and distribute the load. Optimally, the surface of an implant should be able to facilitate bone in‐growth to avoid risks of migration and loosening. However, the newly proposed bioAID uses an UHMWPE fiber surface at the bone implant interface, which, due to its inert chemical characteristics and hydrophobic nature, is less attractive for cells and proteins to attach and facilitate osseointegration.^11^ Therefore, this study aimed to examine the effect of modifying the surface to increase the osteoconductive properties of UHMWPE by altering the hydrophilicity, chemical composition, and surface roughness.

Plasma treatment increased the hydrophilicity of the surface and led to a higher initial cell attachment compared with the untreated group. Multiple in vitro studies have reported similar favorable cellular response when seeded on hydrophilic surfaces compared with hydrophobic surfaces.^18^, ^20^, ^39^, ^40^ Zhao et al. showed a more differentiated phenotype of MG63 osteoblast cells on sand blasted and acid etched titanium surfaces.^40^ In another study of Yamamura et al. it was shown that super hydrophilic treatments of titanium implants increased initial cell attachment, proliferation and differentiation of MC3T3 osteoblast‐like cells.^18^ Similarly, Vrekhem et al. found that plasma treatment of UHMWPE surfaces led to enhanced MC3T3 osteoblast attachment and proliferation.^39^ This view is also supported by Poulsson et al. who showed that human primary osteoblast‐like cells attached and proliferated more on UV/ozone treated UHMWPE surfaces compared with untreated surfaces.^41^ However, in the current study, plasma treatment alone did not increased the ALP activity, which is an often used marker for early stage osteoblast differentiation.^38^ This indicates that only increasing the hydrophilicity of the surface might not be sufficient to support differentiation towards the osteoblastic lineage. Although previous studies have shown increased osteogenic differentiation responses for hydrophilic surfaces, these studies mainly used osteoblast‐like cells from several species which already exhibit osteoblastic markers.^18^, ^20^, ^39^, ^40^, ^41^ Therefore, it could be argued that further differentiation in vitro can be stimulated more easily by hydrophilicity, while for hBMSCs as used here first an osteoinductive stimulus may also be needed.^42^ Osteogenic supplements, being β‐glycerolphosphate, dexamethasone and ascorbic acid, are known to have an osteoinductive effect, however, in this study, these were only added to the medium on day 7. It is generally assumed that MSCs supplemented with osteogenic medium need approximately 14 days to reach the peak in ALP activity, marking the progression of differentiation into the osteoblastic lineage.^43^ Moreover, the rate and extent of osteoblast differentiation initiated by these osteogenic supplements is dependent on the cell density, which could have been different on day 7 when these were added. It should also be noted that in vivo, where this material is intended to be implanted, both osteoblasts and hBMSCs will be present.^44^

Besides plasma treatment, incorporation of hydroxyapatite alone also did not result in significant changes in cell attachment and proliferation. It was expected that the incorporation of HA into the fibers would have a dual effect. On one hand, in vivo and in vitro literature has shown that HA can stimulate osteogenic differentiation by either its geometry, chemical similarities to bone or release of HA ions in the medium.^15^, ^45^, ^46^ On the other hand, incorporation of HA particles into the fiber has led to an increased surface roughness which has also been suggested to be an important factor influencing cellular behavior by acting as an anchor for cellular adhesion.^16^, ^47^, ^48^ Deligianni et al. showed that an increased surface roughness on HA discs led to increased cell adhesion, proliferation and detachment strength.^47^ Likewise, Gittens et al. found that nano and micro scale roughness on titanium substrates improved osteoblast differentiation.^48^ Only few studies have investigated the osteoconductivity of UHMWPE/HA composites in vitro, and mainly as bulk materials, showing a beneficial osteoconductive effect of adding HA compared with pure UHMWPE.^49^, ^50^, ^51^, ^52^, ^53^ Mirsalehi et al. found that UHMWPE/HA composites with increasing weight percentage of HA resulted in enhanced proliferation and differentiation of MG63 cells.^50^ Hermán et al. found a similar trend showing increased initial cell attachment and highest ALP activity for UHMWPE/HA composite with highest (20%) weight percentage HA.^51^ The results in this study did not show an increased cell attachment and ALP activity for samples with HA, but the data did show increasing metabolic activity with increased HA content for both day 2 and day 14. This gives information on which surface cells are more active, for example forming extracellular matrix, but does not indicate to which type of activity. Based on data of cell attachment and ALP activity alone, the current results seem to suggest that the hydrophobic and inert nature of the UHMWPE has a more dominant effect on the cellular response for non‐plasma treated groups. It seems that loading either 10% or 15%HA into the fiber is not sufficient to increase cell attachment and to promote osteogenic differentiation. As also seen by SEM images, loading 10 or 15 wt% HA into the fiber only leads to approximately 3–5 wt% HA exposed at surface, the rest is embedded in the bulk of the fiber.^36^ Therefore, the large polymer surface could interfere with cell attachment. Habibovic et al. also postulated that there is an optimal amount of osteoconductive surface area needed to facilitate bone growth.^26^, ^54^ Another explanation for this discrepancy could be that crystalline HA as used here is the most stable and least soluble ceramic. As a result, crystalline HA can function as an anchor for cells but does not allow for a large increase of calcium and phosphate in surrounding medium to attract cells.^50^, ^55^ Moreover, it is generally stated that HA increases protein adsorption from fetal bovine serum such as fibronectin and vitronectin onto the surface, which facilitates cell attachment.^56^, ^57^ In this study, fetal bovine serum was not added in the medium during the first 2 days of culture. Previous research has shown that in absence of an adsorbed protein layer, HA is a poor substrate for initial cell adhesion and cell spreading.^58^, ^59^ Verdanova et al. reported that in absence of FBS the cells adhere without use of classical focal adhesions that use proteins to anchor them to the surface.^57^ It is hypothesized by Verdanova et al. that cell‐surface contact in absence of FBS is mainly mediated by non‐specific interactions such as van der Waals bonds, hydrogen bonding or charged interactions between polar groups. This might explain the lack of increased cell attachment for 10%HA and 15%HA samples compared with untreated samples, having mainly hydrophobic surfaces. This seems to confirm that surface hydrophilicity because of the plasma treatment has a larger effect on facilitating cell attachment and subsequent cellular processes than only including HA. Nevertheless, it should be noted that cells in absence of serum proteins absorbed from FBS, will synthesize their own matrix to facilitate cell attachment.

Only the groups that contained HA loaded fibers and were plasma treated resulted in both increased cell attachment and upregulated ALP activity. This indicates a dual effect of applying plasma treatment on the HA containing fabrics, resulting in increased hydrophilicity, more HA particles being exposed at the surface (as also confirmed with SEM) and therefore also increased surface roughness. Blatt et al. also found that increasing both surface roughness and hydrophilicity leads to an enhanced effect.^16^

The increased number of cells and metabolic activity present for PT‐HA groups at day 2 in comparison with non‐plasma treated groups disappeared over time when comparing it with data from day 7 and 14. It is hypothesized that this is mainly related to the addition of medium supplements which alter the biological and chemical environment of the cells. It has been stated before in literature that the proliferation and differentiation behavior of hBMSCs is affected by the addition of fetal bovine serum (FBS) resulting in increased proliferation, while β‐glycerolphosphate, dexamethasone and ascorbic acid can stimulate differentiation towards osteoblastic lineage and mineralization.^60^, ^61^ Addition of FBS on day 2 indeed resulted in increased cell proliferation for all groups on day 7. It is generally known that FBS stimulates proliferation, and that fibronectin and vitronectin present in FBS can result in a more appealing surface for cells to adhere, which might explain why the DNA content and metabolic activity became more similar between the groups on day 7 and 14. Schakenraad et al. also found that serum protein coating masks the original surface characteristics, resulting in similar cell spreading and cell growth on different materials.^62^ The addition of osteogenic supplements from day 7 onwards resulted in even more proliferation and thus higher DNA content on day 14. It is generally known that proliferation is related to the synthesis of extracellular matrix, confluency, and cell differentiation. High confluency leads to reduced proliferation and vice versa. Accumulation and maturation of the extracellular matrix results in cells being trapped and embedded, leading to reduced proliferation and increased differentiation. In vivo, this is all tightly regulated by cellular and molecular mechanisms to maintain homeostasis.^63^ In vitro, experimental design decisions, such as initial cell seeding density, are important factors influencing cellular behavior.^61^, ^64^ In this study, only 20,000 cells in a minimal volume of 50 μl were seeded per fabric due to the hydrophobic nature of pure UHMWPE, corresponding to a concentration of 28,000 cells/cm^2^. Cell seeding density can influence cell–cell distance and thereby paracrine signaling that controls cell proliferation and osteogenic differentiation. Previous research has shown that a low cell seeding density led to increased cell proliferation because there is no risk of contact inhibition.^64^, ^65^ The low cell seeding density used could explain the continuous proliferation seen until day 14. Moreover, it might explain the similar amounts of DNA for all experimental groups on day 14, where the non‐plasma treated groups are highly proliferative due to low cell seeding combined with the lower initial cell attachment and thus lower confluency compared with plasma treated samples.

Altogether, it can be concluded that both plasma treatment and incorporation of HA had a positive effect on the osteoconductive potential of the surface. However, a direct relationship between the surface chemistry or topography on cellular behavior is difficult to determine. It is often an interplay between multiple factors that lead to the observed cellular behavior which also makes comparisons with previous studies difficult due to differences in used material, topography, applied culture condition and cell source when using primary cells. To elucidate the role of each surface modification on the increased osteoconductive nature of the surface, quantification of extracellular matrix production and verification of osteoblast differentiation should be expanded.

In this study, it is unlikely that the cells have fully differentiated towards osteoblasts since the DNA content increased over the culture period for all experimental groups while osteoblast differentiation is often coupled with a decrease in cell proliferation.^66^ Moreover, ALP activity can be upregulated on rough surfaces independent of osteoblast differentiation, and it is an early stage osteoblast marker which is less expressed in mature osteoblasts.^38^ Therefore, to gain more insight on the differentiation of hBMSCs towards the osteoblastic phenotype on the different altered surfaces, increased culture period and upregulation of osteoblast markers such as osteocalcin could be identified with for example immunohistochemistry or PCR analysis. On the other hand, such true bone formation may be better evaluated with in vivo implantations.

EDX was used to verify the presence of calcium and phosphorus elements, which are the major components of calcium phosphate apatite, forming the mineral phase of bone. Results showed presence of calcium and phosphorus for all experimental groups. However, EDX is a semiquantitative technique, which can only confirm presence of elements but cannot be used to compare mineral densities. Thus, although more calcium and phosphorus containing spots were detected for PT and/or HA containing groups (not shown), this method cannot elucidate which surface treatment resulted in more mineralization. It is also important to bear in mind that some of the fibers used in this study already contain HA and the addition of β‐glycerolphosphate can induce non‐specific mineral deposition, making the distinction and quantification of cell‐deposited mineralization difficult. To minimize this limitation, only spots in proximity of cells were assumed as cell deposited minerals. As previously mentioned, an increased metabolic activity was measured with increasing HA content that could indicate actively bone depositing cells. Hence, to develop a full picture of the matrix deposition, additional assays that identify collagen synthesis, such as histology, could be valuable.

Since the focus of this study was on the osteoconductive potential of the surface modifications, no extensive surface characterization was included. Nevertheless, an extensive surface characterization was performed on similar fibers as described in the patent publication of Dias et al.^36^ Results confirmed the presence of HA particles with Fourier transform infrared‐attenuated total reflection (FTIR‐ATR) spectroscopy and showed that an increase of HA particles increased the roughness, as measured by the yarn‐to‐yarn coefficient of friction. The patent also describes the effect of the plasma treatment through atomic force microscopy (AFM) and SEM/EDX analysis, verifying the increase of exposed HA particles (3% of area is Ca and Phosphate for 15%HA compared to 16% for PT‐15%HA) and surface roughness. In this study, SEM analysis of the different surfaces also visualize the different densities of HA particles for the different experimental groups, showing the altered roughness. For porous materials it is very difficult to perform contact angle measurements to quantify the hydrophilicity, while it is generally stated that non porous materials are less capable of providing osteoconductivity.^26^ However, addition of medium to the samples clearly visualized the hydrophobic nature of non‐plasma treated surface showing droplet formation, and hydrophilic nature of plasma treated samples immediately absorbing the fluid.

In vitro cell culture studies are used to gain insight into cell adhesion, proliferation, and differentiation on implant surfaces. These parameters are valuable initial indicators for the osteoconductive performance of biomaterials in vivo.^23^ Therefore, this study is useful as initial biological screening of these different surfaces to exclude certain surface modifications and thus reduce the number of in vivo experiments. It can, however, not be fully translated to in vivo performance. In vivo the surface is exposed to heterogenous cell populations and much more complex surrounding fluid. Animal studies remain valuable to provide more accurate data on the dynamics of bone growth on the surface.

## 5 CONCLUSION

Altogether, the current study shows that incorporating HA in UHMWPE fiber together with plasma treatment provides a surface that allows for cell attachment and supports hBMSCs differentiation towards osteoblasts, thereby increasing the osteoconductive potential of the surface compared with untreated UHMWPE fabrics. These findings suggest that this surface modification would be promising for facilitating bone ingrowth on the cranial and caudal surfaces of the bioAID or for any other orthopedic application using UHMWPE fiber at the bone‐implant interface.

## AUTHOR CONTRIBUTIONS

Celien A. M. Jacobs, Esther E. A. Cramer, Aylvin A. Dias, Harold Smelt, Sandra Hofmann, and Keita Ito conceived and designed the study. Esther E. A. Cramer helped in executing the experimental work. Celien A. M. Jacobs obtained all the data presented in this publication. Critical revising of the article was done by all co‐authors. Approval of the submitted version was given by all authors.

## CONFLICT OF INTEREST

Aylvin A. Dias and Harold Smelt work at DSM Biomedical, which owns the licensed patents related to the materials used in this research.

## Data Availability

Data available on request from the authors.
